# A Review on Barrier Properties of Cellulose/Clay Nanocomposite Polymers for Packaging Applications

**DOI:** 10.3390/polym16010051

**Published:** 2023-12-22

**Authors:** Sandile Jali, Turup Pandurangan Mohan, Festus Maina Mwangi, Krishnan Kanny

**Affiliations:** 1 Composite Research Group (CRG), Durban University of Technology, Durban 4000, South Africa; 21502781@dut4life.ac.za (S.J.); mwangif@dut.ac.za (F.M.M.); kannyk@dut.ac.za (K.K.); 2 Department of Mechanical Engineering, Durban University of Technology, Durban 4000, South Africa

**Keywords:** cellulose, clay, barrier properties, permeability, nanocomposite

## Abstract

Packaging materials are used to protect consumer goods, such as food, drinks, cosmetics, healthcare items, and more, from harmful gases and physical and chemical damage during storage, distribution, and handling. Synthetic plastics are commonly used because they exhibit sufficient characteristics for packaging requirements, but their end lives result in environmental pollution, the depletion of landfill space, rising sea pollution, and more. These exist because of their poor biodegradability, limited recyclability, etc. There has been an increasing demand for replacing these polymers with bio-based biodegradable materials for a sustainable environment. Cellulosic nanomaterials have been proposed as a potential substitute in the preparation of packaging films. Nevertheless, their application is limited due to their poor properties, such as their barrier, thermal, and mechanical properties, to name a few. The barrier properties of materials play a pivotal role in extending and determining the shelf lives of packaged foods. Nanofillers have been used to enhance the barrier properties. This article reviews the literature on the barrier properties of cellulose/clay nanocomposite polymers. Cellulose extraction stages such as pretreatment, bleaching, and nanoparticle isolation are outlined, followed by cellulose modification methods. Finally, a brief discussion on nanofillers is provided, followed by an extensive literature review on the barrier properties of cellulose/clay nanocomposite polymers. Although similar reviews have been presented, the use of modification processes applied to cellulose, clay, and final nanocomposites to enhance the barrier properties has not been reviewed. Therefore, this article focuses on this scope.

## 1. Introduction

In the global economy, synthetic polymer plastic materials are produced and made to meet various human needs. The most common application for synthetic plastics is in packaging. Because oxygen and water vapor must be blocked from food and electronic packaging, synthetic polymers are required. Such barriers are required for some food packaging to preserve freshness, prevent diseases and infections, delay food deterioration, and, ultimately, reduce food waste. Because synthetic polymers are primarily made for performance and durability rather than for recycling and degradability, there are significant amounts of discarded polymers in terrestrial and aquatic environments [1]. The main drawbacks of these polymers are their limited recyclability, poor biodegradability, and dependence on fossil fuels [2]. Using synthetic polymers has resulted in environmental pollution, global warming, and the depletion of petroleum reserves. Processes such as hydrothermal treatment and pyrolysis are used to convert plastic wastes into fuels. However, due to toxic gaseous emissions produced during the high-temperature combustion of plastics, its application is limited. Recently, the biodegradation of synthetic plastics using microbes and enzymes has started, but biological treatments are limited due to the difficulty of colonizing microbes and adhering them to the surfaces of plastics [3]. Temporary recovery has come from mechanical recycling, but the volumes are typically too small [1]. Petroleum-based synthetic polymers have become a significant environmental concern, as their levels continue to rise in seas, causing pollution, and they continue to deplete landfill space [4,5]. The development of new bio-based materials with biodegradability, renewability, biocompatibility, and sustainability has increased in demand in recent years [6,7,8].

Because cellulose-based materials are made of aligned nanocellulose, they exhibit an intriguing structural hierarchy. They are also potential substitutes for petroleum-based polymers due to their availability on the earth, renewability, biodegradability, and biocompatibility. Adaptability, broad modification capabilities, and adaptable morphologies contribute to reducing non-renewable natural resources [9]. Cellulose is a linear homopolysaccharide made up of repeating units of anhydro-d-glucose linked by β(1→4) glycosidic bonds, and it has varying degrees of polymerization (DP).

In nature, the DP of cellulose chains is typically around 10,000. However, in some materials, such as native cellulose cotton, the DP can be higher, up to around 15,000 [10]. Three hydroxyl groups are on the monomer, known as the anhydroglucose unit (AGU). These groups give cellulose the capacity to form powerful hydrogen bonds, as well as its crucial characteristics [11].

Cellulose can be extracted from different sources, such as wood [12], agricultural by-products [13], annual plants [13], and marine algae [14]. There are two forms of nanocellulose extracted from plant fibers: cellulose nanofibers (CNFs) and cellulose nanocrystals (CNCs) [15,16]. Many solvent systems and dissolution methods have been developed for cellulose dissolution, such as *N*-methylmorpholine-*N*-oxide (NMMO), LiCl/DMAc, ionic liquids, alkali/urea, NaOH/thiourea aqueous solutions, the TBAF/DMSO system, etc. [17]. However, due to strong hydrogen bond interactions, natural cellulose possesses a high orientation and crystallinity, making it nearly insoluble in ordinary solvents, limiting its applications [18]. Packaging materials made of cellulose have low-water-vapor-barrier properties due to their hydraulic nature, limiting their application in food packaging [19]. Films made of nanocellulose have been reported to lose their oxygen- and water-vapor-barrier properties at high humidity [20,21]. Also, Yu et al. [22] reported that the oxygen permeability of cellulose films increases as the relative humidity increases. Adding fillers, such as montmorillonite, silver nanoparticles, calcium carbonate, and graphene oxide, enhances the water-vapor permeability and mechanical properties of polysaccharide-based matrices [23]. Also, physical and chemical modifications, such as heat treatment, corona modification, plasma treatment, acetylation, periodate oxidation, etc., have been reported to improve properties such as the oxygen and water barrier of the final product [24]. Cellulose and layered silicate clay are among the most abundant polymers and minerals in nature, respectively, both of which show good compatibility with the surface chemistry of polymer composites [25]. Although similar reviews have been presented, the barrier properties of cellulose and nanoclay-based films have not been reviewed for some time despite the abundance of primary research findings. A knowledge gap and a list of unaddressed research questions are presented to aid further work in the field. The objective of this study is to review the work conducted on improving the barrier properties of cellulosic film filled with nanoclay by modifying the cellulose, nanoclay, and final film, which is a gap in the existing literature.

## 2. Preparation of Nanocellulose

The extraction of nanocellulose consists of three stages: pretreatment, bleaching, and nano-sized cellulose isolation. This review summarizes how each of these stages works and their drawbacks.

### 2.1. Pretreatment Methods of Lignocellulosic Biomass

**Figure 1:**
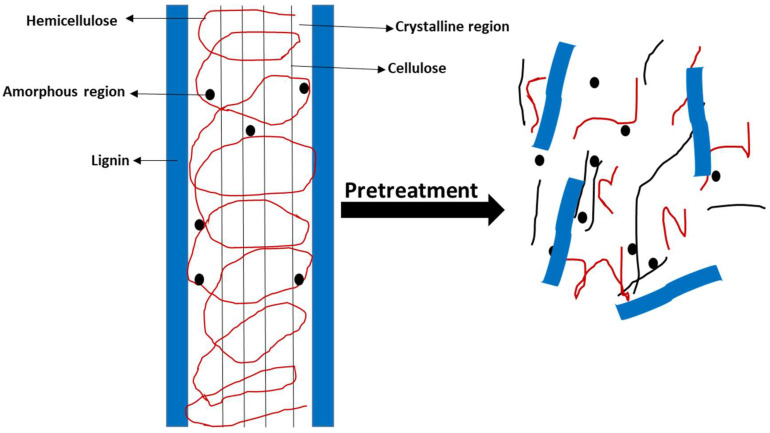
Schematic of pretreatment of lignocellulosic components.

**Figure 2:**
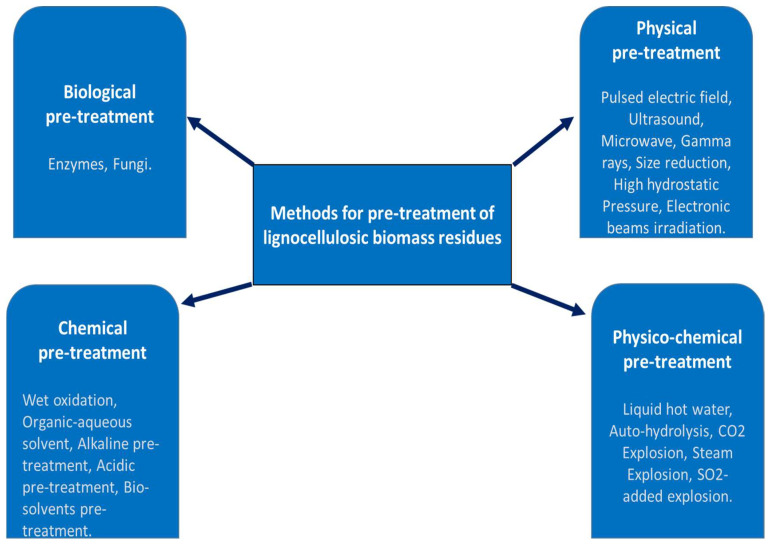
Pretreatment methods for lignocellulosic biomass residues.

Various pretreatment methods are used to remove lignin and hemicellulose by disrupting the complex structure of lignocellulose bonded by non-covalent forces and the cross-linkages of covalent bonds, as shown in Figure 1. These methods are grouped as biological, physical (also known as mechanical), chemical, and physicochemical [26]. The pretreatment methods are illustrated in Figure 2.

#### 2.1.1. Biological Methods

In these pretreatment methods, microorganisms are used to depolymerize the hemicellulose and lignin in lignocellulosic material, such as soft-, brown-, and white-rot fungi [27,28]. Furthermore, bacteria and actinomycetes are also microorganisms that degrade lignin using enzymes such as laccases and peroxidases [29]. Soft and white rots attack lignin and cellulose, while brown rots mainly attack cellulose [30]. This technique has the advantages of consuming low energy, emitting no toxic compounds into the environment, which makes it environmentally friendly, and requiring no chemical recycling [26,28]. The drawbacks of this method are the low hydrolysis rate compared to other technologies [27] and the time consumption [26]. Additionally, improvement in the selectivity for the preferential degradation of lignin by applying non-cellulose or cellulose-deficient white-rot fungi, thereby preserving more cellulose, is still a challenge in fungal pretreatment [29].

#### 2.1.2. Physical Methods

**Figure 3:**
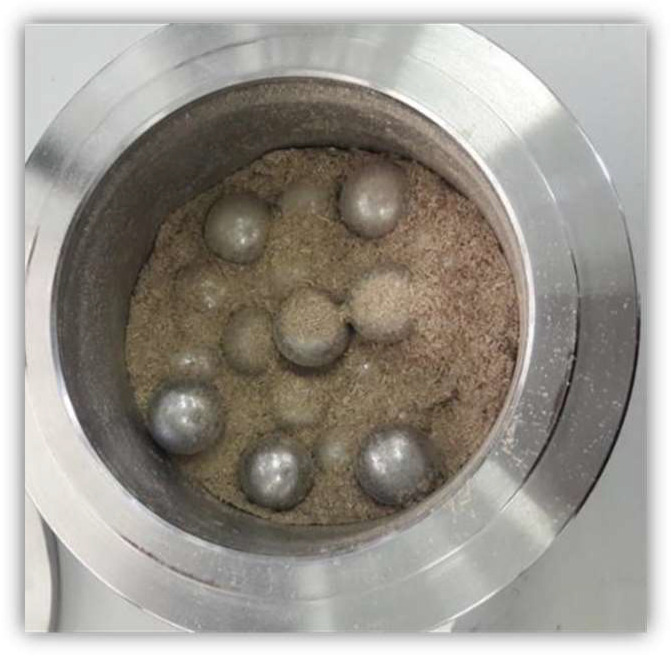
Ball-milling process.

The physical methods comprise mechanical extrusion, milling, ultrasound, pyrolysis, microwaves, and the pulsed electric field. Feedstock materials are heated above 300 °C under shear mixing in mechanical extrusion. This is the most canonical method for the pretreatment of biomass. However, it is a cost-intensive method that is difficult to scale up for industrial purposes because it requires a significant amount of energy [31]. Mechanical grinding, or milling, includes chipping that reduces the biomass size to 10–30 mm, whereas milling and grinding can reduce it up to 0.2–2 mm [32]. Moreover, chipping reduces the mass transfer and heat limitations, while the cellulose crystallinity and particle size are effectively reduced in milling and grinding. Different milling methods are commonly used, like ball mills, shown in Figure 3, centrifugal mills, vibratory mills, extruders, colloid mills, and attrition mills [31]. The particle size of the biomass is decreased with the degree of crystallinity of the cellulose in ball milling. However, energy is consumed, and high power is required for this method [26].

Microwaves are a handy tool for modifying polymer materials by grafting, degrading, and crosslinking them [27]. They are widely used because of their advantages, such as low energy consumption, easy operation, the degradation of the cellulose fraction’s structural organization, and more [28,31]. The drawback of the microwave-heating process is that it is not uniform, meaning that standing waves are formed by the reflection of electromagnetic waves, leading to heat spots on the material [33]. A technique that is relatively new for lignocellulosic biomass pretreatment is sonication. However, it causes small cavitation bubbles, rupturing the hemicellulose and cellulose fractions. In the pulsed electric field, the biomass is subjected to a voltage ranging from 5.0 to 20.0 kV/cm [28,31,34]. However, this technique can be performed in cases that require low energy under ambient conditions [26]. The thermal decomposition of the biomass organic matrix in non-oxidizing atmospheres is known as biomass pyrolysis [35]. The low rate of cellulose decomposition is a disadvantage of this method [36].

#### 2.1.3. Physicochemical Methods

A technique that uses both physical and chemical techniques for breaking the structure of lignocellulosic material is known as steam explosion. In this technique, the material is subjected to a high temperature and pressure for a short duration [37]. However, it has some disadvantages, such as the release of the phenolic compound during the breakdown of lignocellulose. Moreover, during pretreatment, yeast and enzyme inhibitors are generated [38]. Another method similar to steam explosion is the hot-water treatment (also called the hot-compressed-water treatment), which uses pressure up to 5 MPa and a high temperature between 170 and 230 °C instead of steam. This causes hemicellulose to hydrolyze and lignin to be removed, making the cellulose more accessible [31]. However, the extraction of hemicellulose is higher in this technique [38].

Moreover, a lot of energy is required because of the water involved in the downstream processing [31]. Two techniques have been paired by many authors, namely, Ammonia Fiber Explosion and Ammonia Recycle Percolation. In Ammonia Fiber Explosion, the biomass material is subjected to liquid anhydrous ammonia under high pressures and moderate temperatures and is then rapidly depressurized. The disadvantages are the costs associated with recycling and the treatment of the used chemicals [37]. Soaking aqueous ammonia is an ammonia-based pretreatment like Ammonia Fiber Explosion. Ammonia Recycle Percolation is a type of Ammonia Fiber Explosion that treats the biomass with aqueous ammonia in a batch reactor at 30–60 °C, reducing the liquid throughput during the pretreatment process [31].

#### 2.1.4. Chemical Methods

A pretreatment technique used with organic acids (propionic, acetic, and formic acids) and inorganic acids (nitric, phosphoric sulfuric, and hydrochloric acids) is known as acid pretreatment. There are two types of inorganic-acid pretreatment methods, namely, dilute- and concentrated-acid pretreatment [39]. Concentrated acids are not preferred because they are corrosive and must be covered for feasible economic pretreatment [40]. Alkali pretreatment is the most effective method among the chemical pretreatments for breaking the ester bonds between cellulose, lignin, and hemicellulose, and with it, hemicellulose polymer fragmentation is avoided [41]. In alkali pretreatment, alkali compounds are used, such as calcium hydroxide, sodium hydroxide, hydrogen peroxide, potassium hydroxide, ammonium hydroxide, aqueous ammonia, or a combination. However, this pretreatment has drawbacks, such as the incorporation of salts into the biomass or their conversion to irrecoverable salts [40]. Ionic liquids are a new type of cellulose solvent known as green solvents because they do not produce any explosive or toxic gases [39]. In ionic liquids, the hydrogen bonds are broken down during the interaction of the ionic liquid and cellulose-OH, as the breaking of the cellulose molecular chains results in cellulose dissolution [42,43]. However, ionic liquids are still too expensive to be used for biomass pretreatment on a large scale, even though they are an innovative and promising biomass pretreatment technology [38]. Chemicals like acids, salts, oxidizing agents, and alkalis can degrade cellulose, lignin, and hemicelluloses from lignocellulosic waste. An aqueous or organic solvent mixed with inorganic acids (HCl or H_2_SO_4_) can also be used to break the internal lignin and hemicellulose bonds. Furthermore, organic acids, such as oxalic acid, salicylic acid, and acetylsalicylic acid, can also be catalysts [44,45,46].

### 2.2. Bleaching

**Figure 4:**
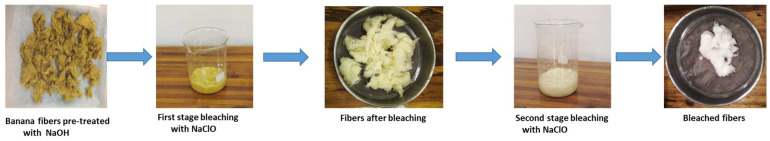
Two-stage bleaching process.

Bleaching is also known as delignification. It is the process of the further removal of the remaining residual cementing material, mainly lignin, from alkali-treated fibers. Bleaching is a necessary additional step because the remaining content of lignin in cellulose after alkali treatment could hinder the extraction of cellulose nanocrystals and lead to a poor wettability surface between the natural fiber and polymer matrix material [16,47,48]. Bleaching is used to degrade hemicellulose and lignin to release cellulose fibers [49]. In bleaching, the solution’s temperature, concentration, time, and pH are the four primary conditions that influence the chemical reactions. Provided that the other factors are kept constant, a greater extent of cellulose is obtained by increasing the reaction time, whereas speeding up the reaction is achieved by increasing the temperature [26]. Examples of agents that are used in bleaching are sodium hypochlorite, hydrogen peroxide, and sodium chlorite [50]. By using the oxidizing agent sodium hypochlorite (NaOCl), it is possible to make lignin removal more efficient [51]. Due to the alkali-resistant linkage that is formed between lignin and hemicellulose after alkali treatment, some studies have used hydrogen peroxide treatment as a precursor to sodium chlorite treatment, which could suppress the lignin removal. In most cases, hydrogen peroxide can break the linkages and delignify lignocellulosic fibers by acting as an oxidizing agent. The bleaching process must be repeated an increasing number of times or stages until the tissues/fabrics are permanently white. Increased bleaching stages are used to increase the cellulose content while removing hemicellulose and lignin, resulting in purer and more separated cellulose fibers than with the previous process [47]. The two-stage bleaching process is shown in Figure 4 below. It can be observed that the fibers after the second stage of bleaching are whiter than those after the first stage.

### 2.3. Methods for Cellulose Nanoparticle Isolation

**Figure 5:**
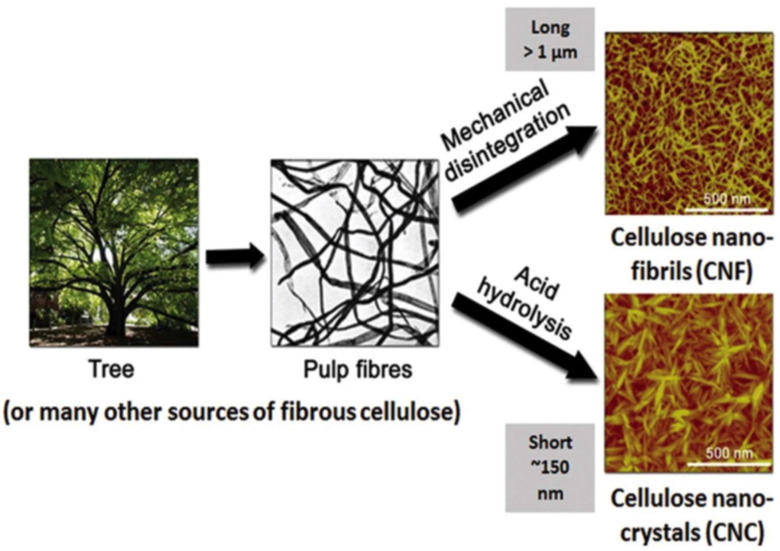
Isolation of cellulose nanoparticles from cellulose source. Reprinted from Ref. [16] (open access).

The simple process of nanoparticle extraction is presented in Figure 5. Fibers are extracted from the source, followed by either acid hydrolysis or mechanical disintegration. Acid hydrolysis is used for nanocrystal isolation, and mechanical disintegration is used for nanofibers. CNCs are rod-like, stiff particles consisting of cellulose chain segments that are nearly perfect crystalline structures. Cellulose nanocrystals exhibit unique liquid crystalline properties and high specific strengths, surface areas, and moduli compared to bulk cellulose, which consists of more significant amorphous fractions [52,53]. These nanocrystals are achieved via several techniques, which are employed to transform cellulose into cellulose nanocrystals. These techniques are grouped as physical hydrolysis (also known as mechanical hydrolysis), enzymatic hydrolysis, and acid hydrolysis. Compared with the mechanical method, the chemical method is better because it produces CNCs with improved crystallinity and reduces energy consumption [54]. However, acid hydrolysis is the most widely explored technique [55,56].

#### 2.3.1. Physical Methods

The mechanical techniques include microfluidization techniques, high-intensity ultrasonic treatments, and cryocrushing, which split the cellulose fibers along the longitudinal axis and help extract the cellulose microfibrils by producing enough shear force [54]. Mechanical methods have also been extensively researched for producing nanocellulose particles as part of the fabrication using enzymatic treatment, acid hydrolysis, oxidation, or a combination. These methods are commonly used to create cellulose nanofibers, which are nanometers or tens of nanometers in diameter and have lengths up to several microns [57]. Among others, ball milling is the most promising method and is widely used for CNC preparation. It is a cost-effective and environmentally friendly technique that is widely preferred over traditional methods (acid hydrolysis) involving concentrated acid. It requires the proper removal of excess acid via dialysis during centrifugation post-processing and is a time-consuming process [58,59]. Some drawbacks include contamination from the grinding containers and medium, usually metal or ceramic contamination, the formation of irregular shapes, and extended cleaning and milling times [60].

#### 2.3.2. Enzymatic Hydrolysis

In enzymatic hydrolysis, the reaction between cellulases and cellulose is involved in degrading the cellulose into fermentable sugars. There are three main cellulose groups: Endo-Glucanase, which attacks the low-crystallinity areas of cellulose fibers and generates free chain ends; Exo-Glucanase, which produces free chain ends that are cleaved to the sugar chains; and b-Glucosidase, which releases glucose from cellobiose [61]. The enzymatic hydrolysis of cellulose offers the potential for higher selectivity, higher yields, milder operating conditions, and lower energy costs than chemical processes [62].

#### 2.3.3. Acid Hydrolysis

Acid hydrolysis is a technique that is currently widely used for the production of nanocellulose. Several acids are used in acid hydrolysis, such as phosphoric, malic, hydrochloric, and sulfuric acids [16,55]. CNCs have recently been produced using hydrochloric, organic, phosphoric, hydrobromic, liquid, gaseous, organic, and inorganic acids. Acid hydrolysis using sulfuric acid is the oldest process, and it remains the most commonly used method for CNC preparation [53]. In acid hydrolysis, amorphous domains regularly distributed along the microfibrils are usually removed using strong-acid hydrolysis. Strong acids can easily penetrate low-order amorphous regions and hydrolyze them while leaving the crystalline regions unaffected [54]. The reaction ends with the surface hydroxyl group of nanocellulose during sulfuric acid hydrolysis, yielding surface sulfate groups, which leads to a negatively charged layer on the nanocrystals. This improves the phase stability of nanocrystalline particles in aqueous solutions even more [55].

## 3. Modification of Cellulose Fibers

Cellulose fiber modification methods are used to improve properties such as the water- and oxygen-barrier properties, mechanical strength, water uptake, and transparency of various food packaging films made of cellulose nanomaterials. There are several modification methods, including esterification, heat treatment, periodate oxidation, TEMPO-mediated oxidation, and so forth. These methods can be classified as physical and chemical methods [24]. This review aims to present the most promising modification processes used to enhance the barriers of cellulose and montmorillonite films.

### 3.1. Physical Modification of Cellulose Fibers

The physical methods include plasma treatment, corona modification, UV light, gamma irradiation, and heat treatment. All these techniques are used to improve the fiber–matrix bonding by reducing the difference between the hydrophobic or hydrophilic properties of the fiber and matrix. Physical treatments appear to be the most environmentally friendly [63].

#### 3.1.1. Heat Treatment

Heat treatment is a physical method that does not affect the chemical composition of the fibers. It is used to improve the fiber strength by increasing the crystallinity index of the fibers [64,65]. Yu et al. [22] found that heat treatment above 100 °C reduced the oxygen permeability at high relative humidity (80% RH) and could be used to drive off water. Also, controlled thermal treatment at various temperatures can be used to create high-oxygen-barrier-performance films. The oxygen permeability of CNF films was reduced by 96 percent after heat treatments, in contrast to films that were not heat-treated [20].

Moreover, Sharma et al. [66] demonstrated that heat treatment reduced the water-vapor permeability by 50% compared to untreated CNF films. From the above studies, it can be seen that heat treatment plays a pivotal role in reducing the oxygen and water properties of cellulose films. Heat treatment reduces the porosity and oxygen permeability [67].

#### 3.1.2. UV Modification

UV radiation promotes carbonyl group formation on the surfaces of natural fibers and wood, improving the fiber surface polarity. Furthermore, UV treatment can cause crosslinking between the surrounding cellulose molecules, which increases the barrier and strength properties of the treated fibers [24].

### 3.2. Chemical Modification of Cellulose Fibers

Chemical treatment aims to activate and modify the fiber structure by using a hydroxyl group, which can change the material’s composition by introducing new elements to the matrix to interact. The use of chemical reagents for fiber modification improves the mechanical properties of the fibers and the strength of fiber-reinforced cement composites, as well as the adhesion between the polymer matrix and fiber surface, by reducing the composites’ water absorption [65]. The chemical modifications include TEMPO-mediated oxidation, periodate oxidation, acetylation, polymer grafting, and more.

#### 3.2.1. TEMPO-Mediated Oxidation

The purpose of TEMPO-mediated cellulose oxidation is to introduce the aldehyde and carboxyl functional groups to the surfaces of the cellulose fibers to improve the mechanical properties. In this method, sodium hypochlorite is added to aqueous cellulose suspensions of sodium bromide and TEMPO. The reaction takes place at room temperature. The aldehyde groups are believed to form covalent bonds with the cationic resin, improving the final characteristics of the composite. The formation of covalent bonds between the microfibrils via hemiacetal and acetal linkages increases the fiber mechanical strength while decreasing the water absorption capacity [24]. Bardet et al. [2] reported an increase in the specific Young’s modulus and tensile strength of TEMPO-oxidized composites compared to untreated TEMPO. However, apart from improving the mechanical properties of the cellulose fibers in free-standing CNC and CNF films, the formation of carboxylic groups on the surfaces of the fibers can improve the composite’s barrier and mechanical properties by reacting with their functional group [68].

#### 3.2.2. Acetylation

Acid catalysts are used in acetylation treatment to graft acetyl groups to the cellular structure of the fibers [69]. During acetylation, an acetyl group reacts with the fiber’s hydrophilic hydroxyl groups to produce esterification, which reduces the fiber’s hydrophilic nature via moisture absorption from the fiber [65]. Because surface hydroxyl groups are involved, the fibers become more hydrophobic after acetylation, increasing their resistance to humidity. This modification improves the final composite’s moisture resistance and mechanical properties [70]. This method increases the fiber’s resistance to increasing humidity, and the final composite consists of improved moisture resistance properties.

#### 3.2.3. Periodate Oxidation

In periodate oxidation, aldehyde groups can cause crosslinking between cellulose chains, improving the film’s barrier properties [24]. Visanko et al. [71] used lithium chloride (LiCl) assisted by sodium meta-periodate (NaIO_4_) oxidation, and the cellulose was converted to dialdehyde. The use of periodate in synthesizing 2,3-dialdehyde cellulose (DAC) with a high degree of oxidation resulted in a more hydrophobic cellulose film with remarkable oxygen-barrier properties, even at elevated relative humidity [72]. Furthermore, researchers prepared butylamino-functionalized CNC films with excellent oxygen-barrier properties at high relative humidity using oxidized cellulose [73].

## 4. Nanofillers

**Figure 6:**
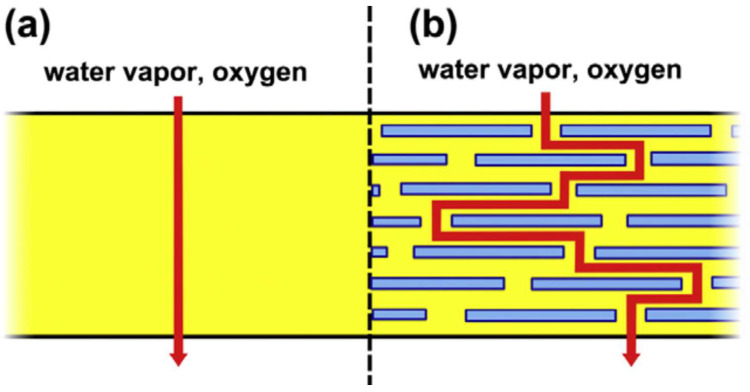
The diffusion of gas molecules in a film made of polymer only (**a**) and a composite film (**b**) (adapted with permission from reference [77]).

Nano-sized materials dispersed in matrix polymers to improve properties such as the mechanical and moisture- and oxygen-barrier properties are called nanofillers [3,74,75]. Jamroz et al. [75] reported that films with one component have high water-vapor permeability and poor mechanical properties, which may hinder their quality and prevent them from being used as packaging materials. Additionally, Abelti et al. [3] reported that it is challenging to use cellulose alone as a packaging material due to its crystalline and hydrophilic nature. The inclusion of inorganic platelets, nanofillers with high aspect ratios, into polymers can significantly alter their barrier properties by altering the diffusion path for gas molecules [76]. Figure 6 below shows the diffusion of gas molecules through the film.

The diffusion of gas molecules can be explained using the figure above. It can be observed that the diffusion pathway in the film with only polymer is perpendicular to the film orientation, whereas, in the composite film, the diffusion pathway of the gas molecules navigates around impermeable nanomaterials. Nano-sized fillers make the tortuous pathway for gas diffusion [77]. There are four types of nanofillers: clay, carbon nanostructure, organic, and inorganic nanofillers. Organic nanofillers include natural biopolymers (such as cellulose and chitosan), whereas inorganic fillers consist of metal oxides (such as ZnO and TiO_2_) and metal (e.g., silver). Carbon nanostructures are categorized into fullerenes, carbon nanotubes, graphene, and nanofibers [75].

### Nanoclays

Nanoclays are inexpensive, have excellent biocompatibility properties, and are widely used in the manufacturing of biodegradable films. They are one of the most preferred and commonly used nanoparticles in packaging development because they are safe for packaging applications due to their natural origin [75,77,78,79]. Bumbudsanpharoke and Ko [80] stated that incorporating a small amount of nanoclay, less than 10 wt%, can substantially improve the polymer’s barrier, thermal, mechanical, and degradation properties. Moreover, Wu et al. [81] reported that nanoclay platelets contributed to oxygen-barrier improvement, even at high humidity. Clays can be classified as montmorillonite (MMT), kaolinite, halloysite, etc. However, montmorillonite is the most widely distributed among clays [23,82]. Montmorillonite is the most commonly used nanoclay, consisting of two tetrahedral silica sheets connected to an eight-sided, edge-divided aluminum oxide sheet [75]. MMT is made of layers (plates) with diameters ranging from 1 to 10 µm and thicknesses varying between 1 and 2 nm, depending on the medium properties. To make MMT hydrophobic, MMT sodium is replaced with ions of cation-active surface-active substances (SASs), which are quaternary ammonium bases or aliphatic amines [82]. Furthermore, Bumbudsanpharoke and Ko [80] mention that nanoclay must be chemically modified to improve its compatibility with the organophilic host matrix.

## 5. Nanocomposite Preparation Methods

Numerous methods are used for the preparation of the nanocellulose-based materials reported. This review aims to present the most promising methods for preparing nanocomposites: the solvent-casting method, the melt intercalation process, and in situ polymerization.

### 5.1. The Solvent-Casting Method

**Figure 7:**
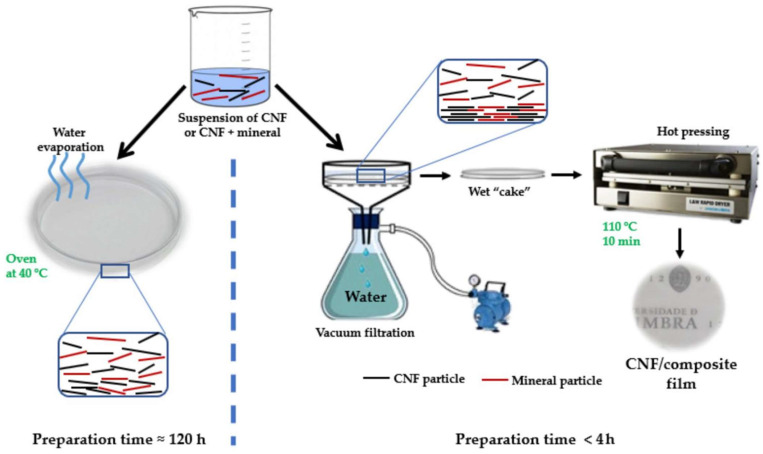
Fabrication of CNF/clay composite films via solvent-casting and vacuum filtration/hot-pressing method. Reprinted from Ref. [16] (open access).

Solvent casting is an easy and widely employed method for preparing nanocomposites. To make cellulose nanocomposites, achieving the good dispersion and distribution of the nanomaterials in the polymer is crucial. Solvent casting has also been used extensively to process CNF-based nanocomposites with various matrices [83]. In order to prepare a homogenous suspension at room temperature, nanocellulose is dispersed within a specific medium (a water or organic medium), which is then mixed with polymer solutions. The mechanical characteristics of the nanocomposites are significantly influenced by this processing method. According to reports, melt-extruded composites fall short compared to nanocomposites created using the solution-casting technique, which is attributed to the nanoparticles’ improved dispersion and the potential for hydrogen bonds to form between the nanofiller and matrix. The solvent-casting technique, an environmentally friendly and low-temperature procedure, uses a small amount of nanofiller to create nanocomposite films of uniform thickness. However, it has some drawbacks: it is limited to the laboratory scale, it requires the high consumption of energy and time, and it is only useful when a very small amount of filler is required [84]. A schematic of NCF/clay composite film fabrication via the solvent-casting and vacuum filtration/hot-pressing method is shown in Figure 7 below.

### 5.2. The Melt Intercalation Process

**Figure 8:**
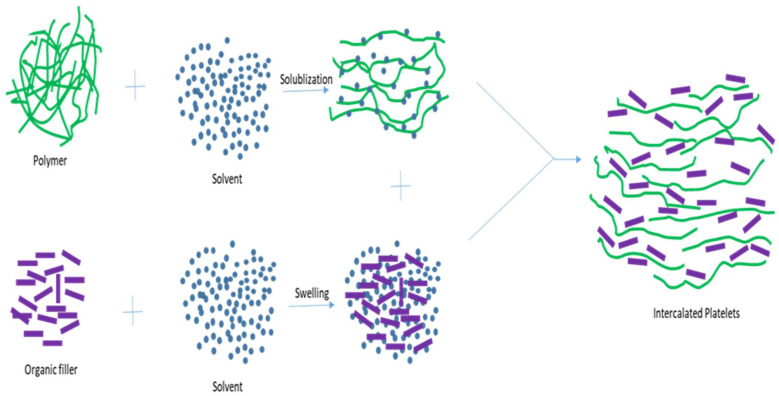
Melt intercalation method.

Vaia, Ishii, and Giannelis published the first report on the melt intercalation process in 1993 [85]. Since then, it has gained a reputation as the most advantageous and valuable method for creating nanocomposites. Compounding tools, like extruders or mixers, are used in the polymer-processing industries to implement this technique [86]. In this top–down approach, nanoparticles and a fused polymer are combined. Nanocomposites are made when the filler and polymer mixture are above the glass transition temperature [84,87]. Polymer chains entering the reinforcement cause intercalation. Either intercalated nanocomposites or exfoliated nanocomposites can be produced, depending on the compatibility (filler and matrix) conditions. The interfacial tension is reduced, and the eco-friendly and straightforward melt intercalation process improves the interactions between the matrix reinforcements. The nanocomposites can be made using polymers unsuitable for solution intercalation, in situ polymerization, or adsorption methods [84,87]. This adaptable approach does not require solvents or chemical reactions [84]. The method is shown in Figure 8 below.

### 5.3. In Situ Polymerization

**Figure 9:**
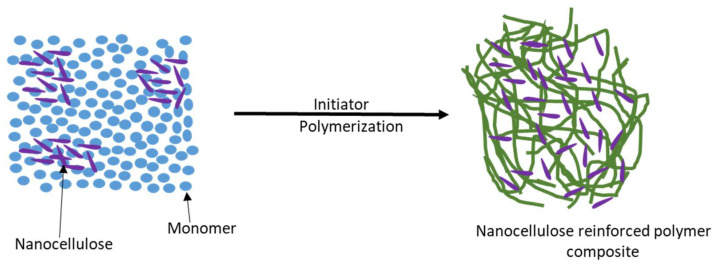
In situ polymerization method.

The first technique used for the synthesis of nanocomposites was in situ polymerization. Figure 9 depicts the in situ polymerization process mechanism. In situ polymerization involves mixing clay with a monomer, and then polymerizing the monomer to lock the exfoliated clay particles into the resulting polymer matrix [87,88]. In situ polymerization has the advantage of achieving the even dispersion of the nanofiller in the polymer by reducing agglomeration. This process is shown in Figure 9 below.

This technique results in a decrease in the moisture absorption behavior and an increase in the biodegradability of the nanocomposites [84]. However, this approach is more limited because it is only applied when liquid monomers are polymerized alongside cellulose in the liquid phase of the polymerization process [89]. This technique is the traditional method for creating thermoplastic nanocomposites. The polymerization reaction for thermosets is started simultaneously by adding a curing agent or peroxide, or it can be started by raising the temperature. The industrial implementation of the in situ polymerization method can significantly speed up the production of cellulosic nanocomposites. This method has produced numerous novel cellulosic nanocomposites with potential applications, including polymethylmethacrylate, polyacrylamide, polyurethane, and polypyrrole [84].

## 6. Barrier Performance

To have adequate food packaging, it is essential to prevent moisture and oxygen transmission between food and the environment. The exchange of moisture and oxygen impacts the shelf life, taste, odor, appearance, marketability, and quality of packaged food. As a result, the barrier properties of food packaging materials play a pivotal role in extending and determining the shelf lives of packaged foods [19,79,90]. The barrier requirements vary depending on the product to be packaged. Some of the barrier requirements for specific food products are shown in Table 1. In packaging, oxygen can degrade the food quality, and food packaging materials with lower oxygen permeability (OP) values are preferred [91]. Nanocomposites are continuously improved with additives/fillers [92]. Various clays have been used as fillers to improve the oxygen- and moisture-barrier performance [93]. 

Several approaches have been applied to improve the barrier performance of cellulosic films/nanocomposites by modifying the cellulose, nanoclay, or final film. This modification can be either chemical or physical. Bardet et al. [2] prepared a cellulosic film reinforced with montmorillonite. In this study, the cellulose was modified using TEMPO-mediated oxidation with water containing NaBr (2.5 mmol per gram of nanocellulose) and TEMPO (0.1 mmol per gram of nanocellulose)) under vigorous stirring. This process was conducted at room temperature. The film was prepared by mixing MMT with an NCF suspension using solvent casting. The resulting film was subjected to heat treatment at 140 °C for 2 h in a thermostatic oven. This typical film was reported to exhibit an excellent oxygen permeability of 1 ${cm}^{3}$ μ${mm}^{-2}$ ${day}^{-1}$ ${kPa}^{-1}$ at 50% RH. However, the film showed accelerated water uptake above 50% RH. Notably, among the chemical modification processes, TEMPO-mediated oxidation is used, and heat treatment is used among the physical optimization processes.

Cellulose/montmorillonite composite films with lower oxygen permeabilities than those of commercial ethylene−vinylalcohol copolymer films were prepared by Wu et al. [81]. These films had noticeably lower OPs under dry conditions. The composite films of this study were prepared via the solvent-casting method, with the cellulose TEMPO-oxidized via the TEMPO/NaBr/NaClO system in H_2_O and then mixed with mechanically treated MMT. The mechanical treatment of MMT was performed by dispersing it in water and stirring it for 1 h using a magnetic stirrer. Different cellulose-to-MMT ratios were used. The composite film with a 50:50 cellulose/MMT ratio had a lower OP value of 0.0008 mL μm m^−2^ day^−1^ kPa^−^^1^. However, at 50% RH, the OP increased to 0.2 mL μm m^−2^ day^−1^ kPa^−1^. It can be noted that the barrier performance of this film is greater than that of ethylene−vinylalcohol films only at 0% RH. However, the barrier property is lower than ethylene at 50% RH. Upon the application of optimization processes, the cellulose was chemically modified (TEMPO-mediated oxidation) and the MMT was mechanically modified.

Also, a study conducted by Yang et al. [94] obtained results similar to those obtained by Wu et al. [81], showing the better barrier performance of cellulose/montmorillonite compared to a commercial ethylene–vinylalcohol copolymer at 0% RH. Yang et al. [94] used LiOH/urea/H_2_O to disperse MMT. The mixture was stirred for 2 h and homogenized before adding cellulose. The solvent-casting method was used. Neither chemical nor physical modification was used. The oxygen permeability values were as follows: below 0.0005 mL μ${mm}^{-2}$ ${day}^{-2}$ ${kPa}^{-1}$ at 0% RH; 0.58 mL μ${mm}^{-2}$ ${day}^{-1}$ ${kPa}^{-1}$ at 50% RH; and 5.9 mL μ${mm}^{-2}$ ${day}^{-1}$ ${kPa}^{-1}$ at 75% RH. It was noted that as the humidity increased, the oxygen permeability increased. It was further noted that the water vapor also increased with the increased humidity. The significant increase in the OP was because the hydrophilic cellulose molecules resulted in a moisture content greater than 10% at 50% RH. This was mentioned to promote the diffusion and dissolution of oxygen molecules in the water molecules absorbed in the film [95]. Of note, none of the chemical or physical modification methods were used.

The water-vapor permeability of a cellulose/montmorillonite composite prepared by adding different amounts of MMT in 0.2 wt% nanocellulose suspensions was investigated by Garusinghe et al. [90]. The mixing time was about 3–18 min, and high-pressure homogenization was used to prepare the original suspension before the film formation. The resulting films had 9.1, 16.7, 23.1, 28.6, 33.3, and 37.5 wt% MMT. A reduction in the WVP as the clay content increased was observed, with a minimum value of 13.3 ± 2.0 g μm/$m^{2}$ day kPa reached at 16.7 wt% MMT loading, followed by an increase in the WVP. In comparison, composites prepared with high-pressure homogenization showed minimum WVPs (6.3 ± 1.5 g μm/$m^{2}$ day kPa) at a higher MMT loading of 23.1 wt%. The decrease in the WVP was said to be due to the tortuous path caused by the MMT platelets perpendicular to the diffusion path, following an increase in the WVP after the value was reached, attributed to the more water absorbed by the hydrophilic MMT in the composite, which opened more pores [90]. Notably, no chemical or physical modification processes were used.

The permeation of gases in a composite film comprising nanocellulose/montmorillonite was also carried out by Wang et al. [96]. The film was prepared using the solvent-casting method, where cellulose was dissolved in a LiOH/urea/$H_{2}O$ solvent, and then MMT was added. Cellulose/MMT hydrogels were soaked in glycerol, and plastic fabrication was performed via hot pressing at 110 °C. The permeation of gases like N_2_, H_2_, CO_2_, and CH_4_ decreased with an increase in the MMT content. Of note, the gas barrier of these nanocomposites was better than those of commonly used synthetic polymers, like polystyrene, polyethylene, and polypropylene. Farmahini-Farahani et al. [97] used the same solvent used by Wang et al. [96] to prepare cellulose/MMT nanocomposite films and investigated their water-vapor resistance. In this study, Cloisite Na^+^ (Na-MMT) was added to LiOH/urea/$H_{2}O$ and stirred and filtered before being pre-cooled. The resulting hydrogel was soaked in acetone and dried at room temperature to obtain the composite film. The addition of nanoclay improved the water-vapor permeability. In the study, nanoclay was used without modification, as well as cellulose.

A study by Ferfera-Harrar and Dairi [98] showed a commonly observed relationship between a decrease in the WVP and an increase in the MMT loading. Cellulose acetate was used (i.e., cellulose modified via acetylation). Moreover, the used MMT was organo-modified with gelatin or chitosan. An acetic/water solvent was used. The film with 5 wt% MMT presented a WVTR value of 84 ± 2 $g_{water}$/$m^{2}$.day, whereas the neat film showed a value of 128 ± 3 g_water_/$m^{2}$.day. A remarkable improvement in the WVTR via the addition of clay was reported. At the time of their article, Mahmoudian et al. [99] claimed that no study of cellulose/montmorillonite nanocomposite films prepared in ionic liquid had been reported. Thus, in their study, an ionic liquid called 1-butyl-3-methylimidazolium chloride (BMIMCl) was used as a solvent. They reported that carbon dioxide and oxygen were reduced by 31% and 33%, respectively, by incorporating 6 wt% MMT. The oxygen and carbon dioxide values for the permeabilities were 0.76 ± 0.11 × $10^{-18}$ $m^{3}$ m/$m^{2}$ s Pa and 1.26 ± 0.06 × $10^{-18}$ $m^{3}$ m/$m^{2}$ s Pa, respectively, at 6 wt% MMT loading. None of the chemical or physical modification processes were used in the study.

The effect of nanoclay on the oxygen and water-vapor properties at 50% and 80% RH were studied by varying the lithium-exchanged vermiculite content in the preparation of biohybrid films in a study by Aulin et al. [100]. The films were fabricated using the solvent-casting method, where a 0.2 wt% NFC dispersion was added to a 0.2 wt% VER dispersion. After high-pressure homogenization, films were formed upon solvent evaporation at room temperature. Films with 0, 5, 10, and 20 wt% nanoclay contents were developed. At both relative humidities, the 20 wt% nanoclay film showed lower permeability than the neat cellulose film. The oxygen permeabilities were 0.07 cm^3^ mm^−^^2^ d^−^^1^ kPa^−^^1^ and 1.5 cm^3^ mm^−^^2^ d^−^^1^ kPa^−^^1^ at 50% and 80% RH, respectively. Also, the WVPs were 0.7 ng m m^−^^2^ s^−^^1^ kPa^−^^1^ and 21.2 ng m m^−^^2^ s^−^^1^ kPa^−^^1^ at 50% and 80% RH, respectively. The used nanoclay was modified via repeated cationic exchange with delaminated citrate ions and lithium salts, followed by mechanical shearing.

**Figure 10:**
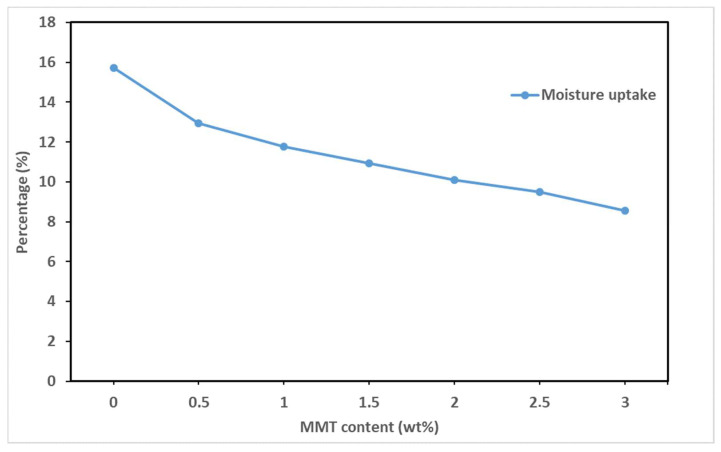
Moisture uptake versus MMT content.

Gibbsite nanoclay was used by Sethi et al. [21] to prepare a hybrid cellulose film via the solvent-casting method. The results showed a factor of 36 lower oxygen permeability of gibbsite/cellulose film compared to neat cellulose film. These results were obtained at 50% RH and were said to be in good agreement with those of Liimatainen et al. [101] and Aulin et al. [100] for CNF/clay hybrid films. Yao et al. [102] conjugated dopamine (DA) to cellulose nanofibrils (CNFs) and added it to an MMT dispersion in water. The solvent-casting method was employed. It was observed that the composite film with DA-CNF/MMT had a lower oxygen transmission rate at 50% and 95% RH than the CNF/MMT film. This resulted from the interfacial adhesion between the CNFs and MTM. An investigation of the effect of the modification of nanoclays on the water-vapor property was carried out by Peighambardoust et al. [103]. The study used Cloisite 30B and MMT nanoclays to make cellulose/nanoclay composite films. These clays were modified with Cu and Ag ions. It was observed that the Cloisite 30B had more effect on the WVP than the MMT. Also, the Cu-modified Cloisite 30B clay had a higher reduction in the WVP compared to the Ag-modified clay. SEM also revealed this, where Cloisite 30B was more compatible with the cellulose matrix and created a more homogeneous surface morphology. Also, in a study conducted by us, the moisture uptake of film prepared by incorporating MMT (Cloisite 30B) into the cellulose films was improved by 46%. The results are shown in Figure 10 below.

This reveals that the modification of the nanoclay before use for film fabrication can enhance the barrier of the final film. Moreover, it is noticeable that limited studies have employed this method to improve the barrier properties. The modification of cellulose fibers via acetylation, periodate oxidation, and more have been reported to strengthen the barrier performance. However, their application in film fabrication is limited. One or more promising methods could produce the best-performing film, even at high humidity. These studies are summarized in Table 2 below.

Recently, Garusinghe et al. [90] mentioned that few studies on montmorillonite and nanocellulose films have focused on the water-vapor permeability. Furthermore, Saedi et al. [24] stated that the modification of cellulose nanomaterials has been reported, but its importance in barrier improvement has not been discussed. Therefore, this presents a research gap, as there are numerous cellulose nanomaterial modification processes, especially those that improve the film barrier properties. According to the literature above, the problem can be identified as a significant increase in the permeability of cellulosic film at high humidity. This was also mentioned by Yang et al. [94]. A chemical modification process, namely, acetylation, was reported by Shojaeiarani et al. [70] to improve the film barrier resistance to increased humidity. Moreover, periodate oxidation was also reported by Plappert et al. [72] to result in a film with excellent oxygen-barrier properties, even at high humidity. The effect of combining two or more of the mentioned modification processes on the barrier properties of cellulose/montmorillonite has not been reported. Ionic-liquid solvents are the least explored for preparing these films for water-vapor and oxygen properties.

## 7. Processing Features and Challenges

The features of nanocellulose and polymer nanocomposites are highly dependent on the preparation process used, as discussed in this article [104]. For example, solvent casting results in a rather homogeneous concentration increase due to slow solvent evaporation, which brings the CNF fibrils and clay rods or plates closer together, ultimately inducing aggregation and interactions. In contrast, vacuum filtration quickly removes the solvent with the aid of a vacuum, causing the fibrils to concentrate only at the bottom, forming a densely packed network, while the supernatant dispersion stays close to the initial conditions. Composites prepared via vacuum filtration exhibit lower porosity and higher tensile strength [105]. Poorly distributed and dispersed nanofillers within the polymeric domain structure remain a major issue with nanocellulose and polymer nanocomposites [106]. Despite the good features of nanocellulose, the manufacturing costs are still not competitive with those of petroleum-based polymers [86]. This means that cellulose/clay composite production may be expensive, limiting its industrial application. The first attempt to create cellulose fiber CNF/kaolinite nanocomposites on a pilot-scale sheet former was presented in 2018 by Castro et al., demonstrating the method’s potential for industrialization [105]. Although there are several applications of cellulose films in industries, the industrial use of cellulose/clay composites specifically has not been reported. Solvent casting is the commonly used method for the production of these composites, but it is limited to the laboratory scale, which is a drawback. Therefore, more work is needed to transform these materials for large-scale and industrial applications.

## 8. Conclusions and Future Perspectives

Cellulose/nanoclay composite film has shown considerable potential for use as a barrier material. It has been found that the film demonstrates excellent barrier properties at low relative humidity but a loose barrier at high humidity. Modification processes have been used to counteract the moisture sensitivity of the film. Comparing the studies of Wu et al. [81] and Yang et al. [94], the oxygen permeabilities at 50% RH were 0.2 mL μm m^−2^ day^−1^ kPa^−1^ and 0.58 mL mL μm m^−2^ day^−1^ kPa^−1^, respectively. Wu et al. [81] used TEMPO-mediated oxidation and mechanically modified the clay, which resulted in a 25.6% reduction in the oxygen permeability compared to that obtained by Yang et al. [94]. It has been revealed in the literature that different clay optimization processes result in different barrier properties of the final composite [103]. This review article focuses specifically on the barrier properties of cellulose/montmorillonite films, and the use of physical and chemical modification processes that have been reported to enhance them. Limited studies have been conducted specifically on these types of films to improve their oxygen and vapor properties, and very few studies have employed one or more modification processes for the improvement of these typical films. More research is needed on the approach of using optimization processes, which can be performed on cellulose, clay, or a combination of both. This review article also highlights the solvents used for these nanocomposite fabrications and shows that few solvents have been explored. For instance, there are various ionic-liquid solvents, such as 1-Allyl-3-methylimidazolium chloride, 1-ethyl-3-methylimidazolium acetate, and more, which have been used for film production. However, these solvents have not been reported in the preparation of cellulose/clay materials for barrier application. In the future, the development of high-barrier bio-based cellulosic materials will be the trend. More research is needed on the modification of nanoclay, cellulose, and film for barrier improvement. With more research on the functional modification of cellulose, cellulose-based packaging, and particularly nanocellulose-based packaging, will be widely used in high-barrier film, hydrophobic packaging, modified-atmosphere packaging, and more [107].

## Figures and Tables

**Table 1:** Barrier properties required for some specific food products.

Product Name	Oxygen Permeance (cm^3^ STP/m^2^·day·Pa) 23 °C	WVTR (g/m^2^·day) 23 °C	Ref.
**Dairy products**	6 ×$\;{10^{-}}^{4}–5\;\times$ 10^−2^	0.2–8	[86]
**Nuts, snacks, chips**	1.6 ×$\;{10^{-}}^{6}–9.6\;\times$ 10^−5^	0.093–3.0	[86]
**Hard cheese**	8.6 ×$\;{10^{-}}^{4}–3.45\;\times$ 10^−3^	50	[86]
**Meat and meat-based** **products**	2 ×$\;{10^{-}}^{4}–1\;\times$ 10^−1^	2–100	[86]
**Retorted food**	5.9 ×$\;{10^{-}}^{6}–5.0\;\times$ 10^−5^	0.40–7.6	[86]
**Fruits, vegetables,** **fresh salads**	1 × 10^−1^–2	10–4000	[86]
**Fats**	6.8 ×$\;{10^{-}}^{5}–8.0\;\times$ 10^−4^	5.2–9.2	[86]

**Table 2:** Different solvents and cellulose chemical and physical modifications for cellulose/montmorillonite films used in previous studies for barrier performance.

S. No	Solvent	Methods	Cellulose Chemical Modification	Cellulose/Film Physical Modification	Nanoclay Modification	Key Findings	Ref.
**1**		Solvent casting	TEMPO-mediated oxidation	Heat treatment		Excellent oxygen permeability at 50% RH	[2]
**2**		Solvent casting	TEMPO-mediated oxidation		Mechanical	Oxygen permeability is lower than ethylene−vinylalcohol at 0% RH	[81]
**3**	LiOH/urea/H_2_O	Solvent casting				Oxygen permeability is lower than ethylene−vinylalcohol at 0% RH	[94]
**4**		Solvent casting				Decrease in WVP as clay content increases	[90]
**5**	LiOH/urea/H_2_O	Solvent casting				Permeation of gases like N_2_, H_2_, CO_2_, and CH_4_ decreased with increase in MMT content	[96]
**6**	LiOH/urea/H_2_O	Solvent casting				Improved water-vapor permeability via the addition of nanoclay	[97]
**7**	Acetic acid/water	Solvent casting	Acetylation		Nanoclay organo-modified with gelatin or chitosan	Decrease in water-vapor transition rate for 5 wt% MMT film compared to neat cellulose film	[98]
**8**	BMIMCl	Solvent casting				33% reduction in oxygen and 31% in carbon dioxide permeability via 6 wt% MMT loading	[99]
**9**	Solvent casting				Nanoclay modified via cationic exchange with delaminated citrate ions and lithium salts	20 wt% nanoclay film showed lower permeability compared than neat cellulose film	[100]
**10**	Water	Solvent casting			Nanoclay modified with Cu and Ag ions	Cu-modified Cloisite 30B clay had a higher reduction in WVP compared to Ag-modified clay	[103]
**11**		Solvent casting				Composite film with DA-CNF/MMT had lower oxygen transmission rate	[102]
**12**		Solvent casting				Gibbsite/cellulose film had a factor of 36 lower oxygen permeability compared to neat cellulose	[21]

## Data Availability

Not applicable.
